# Advances in Food-As-Medicine Interventions and Their Impact on Future Food Production, Processing, and Supply Chains

**DOI:** 10.1016/j.advnut.2025.100421

**Published:** 2025-04-04

**Authors:** Thijs Defraeye, Flora Bahrami, Tobias Kowatsch, Simon Annaheim, Marjolijn CE Bragt, René M Rossi, Michael Greger

**Affiliations:** 1 Empa, Swiss Federal Laboratories for Materials Science and Technology, Laboratory for Biomimetic Membranes and Textiles, St. Gallen, Switzerland; 2 Food Quality and Design, Wageningen University & Research, Wageningen, The Netherlands; 3 ARTORG Center for Biomedical Engineering Research, University of Bern, Bern, Switzerland; 4 Institute for Implementation Science in Health Care, University of Zurich, Zurich, Switzerland; 5 School of Medicine, University of St. Gallen, St. Gallen, Switzerland; 6 Department of Management, Technology, and Economics at ETH Zurich, Centre for Digital Health Interventions, Zurich, Switzerland; 7 Wageningen Food and Biobased Research, Wageningen University & Research, Wageningen, The Netherlands; 8 NutritionFacts.org, Takoma Park, MD, United States

**Keywords:** food-is-medicine, fruit, vegetables, postharvest, supply chain, precision nutrition

## Abstract

Food-as-medicine (FAM) is an emerging trend among medical doctors, health insurers, startups, and governmental public-health and nongovernmental organizations. FAM implies using food as a part of an individual’s health plan to prevent or help treat acute and chronic health conditions and diseases. We highlight trends and hurdles in the FAM intervention pyramid. Our viewpoint is to indicate how interventions might change the future demand for specific food groups, their transport in supply chains, and the technologies used to process them. On the basis of national guidelines, dietary interventions can help to prevent and treat many diseases, including cardiovascular disease, cancers, type 2 diabetes, and obesity. FAM R&D and services offer more individualized treatments. This is challenging given the interindividual variability and complexity of the body’s response to food and related factors, such as dietary habits, genetics, lifestyle, and biosphere. Quantifying health improvements is essential to prove the added value of more individualized FAM interventions compared with adopting a general healthy diet. It is unclear which level of individualization of interventions produces the largest health benefits at the lowest costs for the patient, healthcare system, and climate. FAM interventions can support and complement conventional medical treatment. They will require a shift to producing more health-promoting foods, including whole foods, minimally processed foods, and selected processed foods. The food processing industry and supply chains must adapt to these new scenarios. Auxiliary technologies and methods are enablers, including delivery services, wearable technology, health-monitoring apps, and data-driven consumer behavior analysis.

## Introduction

The food we eat impacts our physical and mental health and well-being. In the United States alone, diet-related diseases (cardiovascular diseases, hypertension, cancer, and diabetes), obesity, and food insecurity cost an estimated 1.1 trillion [1], namely 604, 359, and 146 billion USD, respectively. Other studies reported 50 billion USD in diet-related costs for cardiometabolic diseases in the United States per year, or 300 USD per capita [2]. Worldwide, unhealthy diets account for an estimated 3–150 EUR of healthcare costs per capita [3]. Furthermore, 1 of every 5 deaths globally (11 million people) in 2017 was attributed to an unhealthy diet, leading to 255 million disability-adjusted life years [4]. About half of these deaths happen in adults younger than 70 y. According to the Global Burden of Disease Study [4], the 5 main causes of diet-related mortality globally are a high sodium intake and a low intake of whole grains, fruit, nuts/seeds, and vegetables. These diet-related deaths are induced by cardiovascular disease (89%), various types of cancer (8%), and type 2 diabetes (3%). These deaths are partly caused by food insecurity, meaning people who do not get enough food or do not get enough of the appropriate food. Another cause would be overeating and unbalanced meals, but many other aspects are responsible for such diet-related mortality. The associated costs for individuals and healthcare systems are huge. The aging population will amplify these costs in the future, as more older people will likely require more healthcare.

Besides other lifestyle factors such as daily physical activity, changing the diet or its parts has a high potential to prevent several chronic and acute diseases or health conditions and decrease mortality [5,6]. In specific cases, food can also help treat these conditions and support conventional medical treatment [7]. Nevertheless, nutrition care has not yet been fully adopted as a part of patient-centered healthcare [8]. However, the role of diet in human health has been a cornerstone in many cultures through traditional medicine for centuries (Chinese, European, or Ayurvedic medicine). The rebranded concept for health management—food-as-medicine (FAM)—is now an emerging trend [[9], [10], [11], [12], [13], [14]]. In FAM, however, new components have been integrated by deploying the latest, mainly digital, technologies.

**FIGURE 1:**
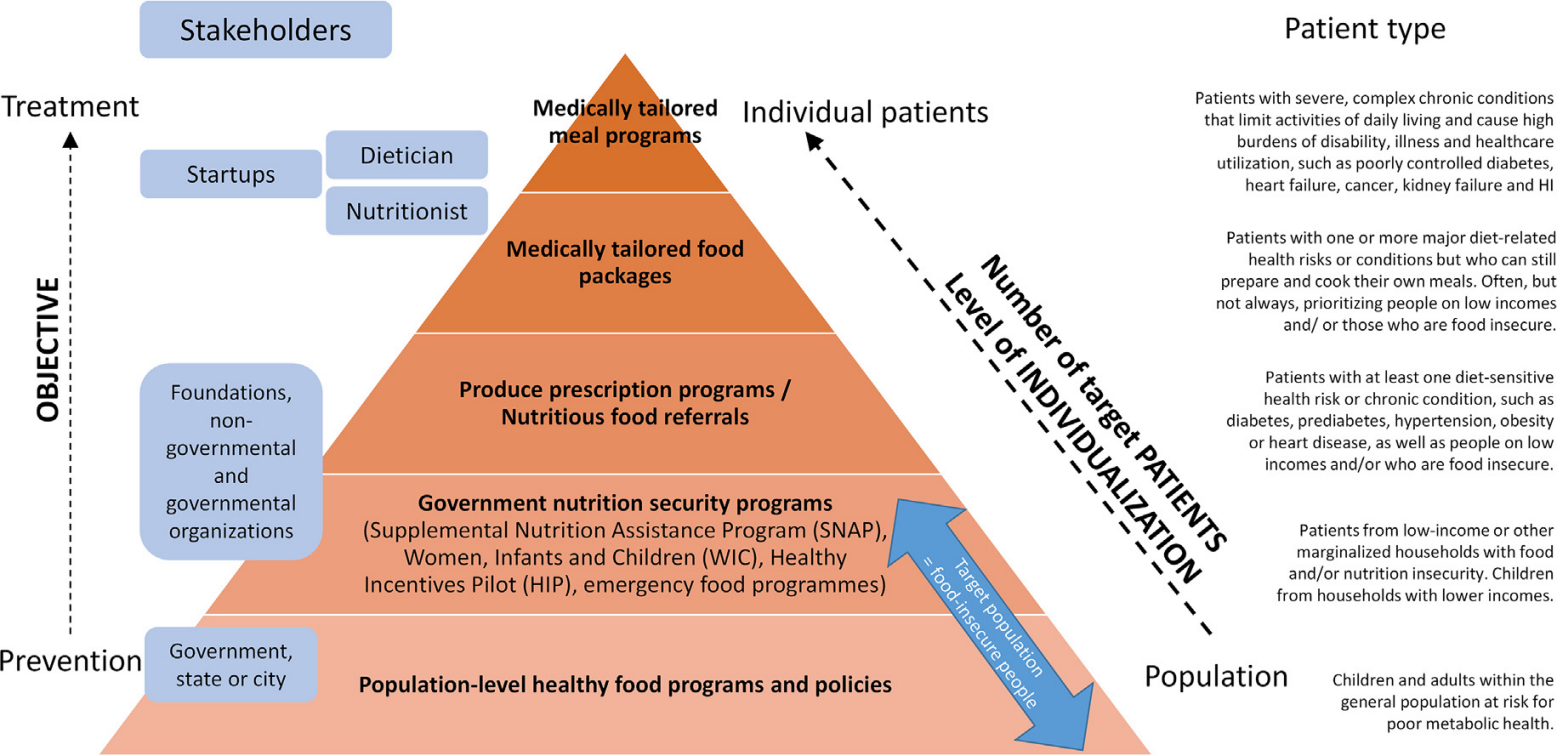
Food-as-medicine intervention pyramid (adjusted from [11,12]).

FAM implies using food as a part of an individual’s health plan, primarily focused on patients with a disease or a predisposition to a disease or health condition. It aims to help prevent or manage diseases and health conditions, thus supporting conventional medical treatments. FAM thereby aims to *1*) promote health, improve overall well-being, and reduce risk of developing chronic diseases [15], *2*) reduce healthcare and medication costs for the public healthcare system and the patient, and *3*) lower the healthcare system’s utilization, including reduced emergency department visits. FAM interventions rely, among others, on whole food products or minimally processed foods, which are then further processed into meals. Next to these foods, preprocessed foods fortified with functional compounds are also deployed within the concept of FAM. An example is plant-based proteins enriched with vitamins. We do not consider food supplements administered separately from a meal or food product to be part of FAM. FAM is targeting different types of interventions for different populations. FAM is therefore implemented in several hierarchical levels. This hierarchy is often represented in a FAM pyramid (Figure 1), which is discussed in detail in hierarchical levels of FAM section.

FAM recently attracted massive attention from medical doctors, health insurers, startups, governmental public health and nongovernmental organizations. The drivers that enabled this FAM trend are recent developments in digital platforms and technologies, data science, and artificial intelligence (AI), food functionalization and fortification, wearable-based health monitoring, and consumer demand for more individualization of nutrition, medicine, and healthcare. Different stakeholders want to solve the question of how to optimize our food to treat and prevent health conditions and complement or partly replace conventional medicine. They also promote patient self-monitoring and digital advice to initiate behavior change and identify the health complications that benefit the most from this approach. How far should we go down the rabbit hole in developing personalized FAM interventions to significantly impact human health for individuals and entire communities or populations? The answer will impact the future food production, health monitoring, data infrastructure, supply chains (SCs), and the food processing industry.

In this study, we aim to help solve these questions. We first show how diet and food can help prevent and contribute to treating lifestyle diseases. We define what FAM is, give an overview of the different hierarchical levels of interventions, and discuss the current trends. We present an extended FAM pyramid. We highlight problems and hurdles that can occur with further deploying FAM. A particular focus of this work is that we indicate the potential impact of FAM and dietary guidelines on food SCs, food production, and food processing technology, which are essential for enabling FAM interventions. We evaluate which FAM interventions have the highest potential, focusing on which level of individualization we need to target.

## Examples on How Diet and Individual Foods Can Help Treat and Prevent Acute and Chronic Health Problems and Diseases

### Context

To lay a foundation for the concept of FAM, we first provide examples of how diets and foods can help to treat or prevent acute and chronic diseases and health conditions. The knowledge base of studies is extensive and is detailed in the following sections. The reasons are *1*) a large number of health conditions, including cardiovascular diseases, various types of cancer, diabetes, obesity, hypertension, or osteoarthritis; *2*) many diets, such as omnivorism, veganism, pescetarianism, ovo-vegetarianism, lacto-vegetarianism, ovo-lacto-vegetarianism; *3*) a multitude of individual foods that have an impact on each of these health conditions. Several combinations have been tested between diets or foods for health conditions. Also, patients with different baseline conditions have been evaluated, including a different baseline diet, race/ethnicity, socioeconomic background, or chronic disease status [6]. As a result, there are many studies on the impact of a single diet or food on a single disease, with sometimes even contradictory findings.

Therefore, we focus on meta-analyses and systematic reviews for specific health conditions or diseases because the entire body of knowledge is too vast to consider all diseases and foods. These studies typically do screening and prioritize randomized, double-blind, placebo-controlled trials while excluding nonviable studies, including nonrandomized control trials, ineligible populations, and funder bias.

### Dietary guidelines

**FIGURE 2:**
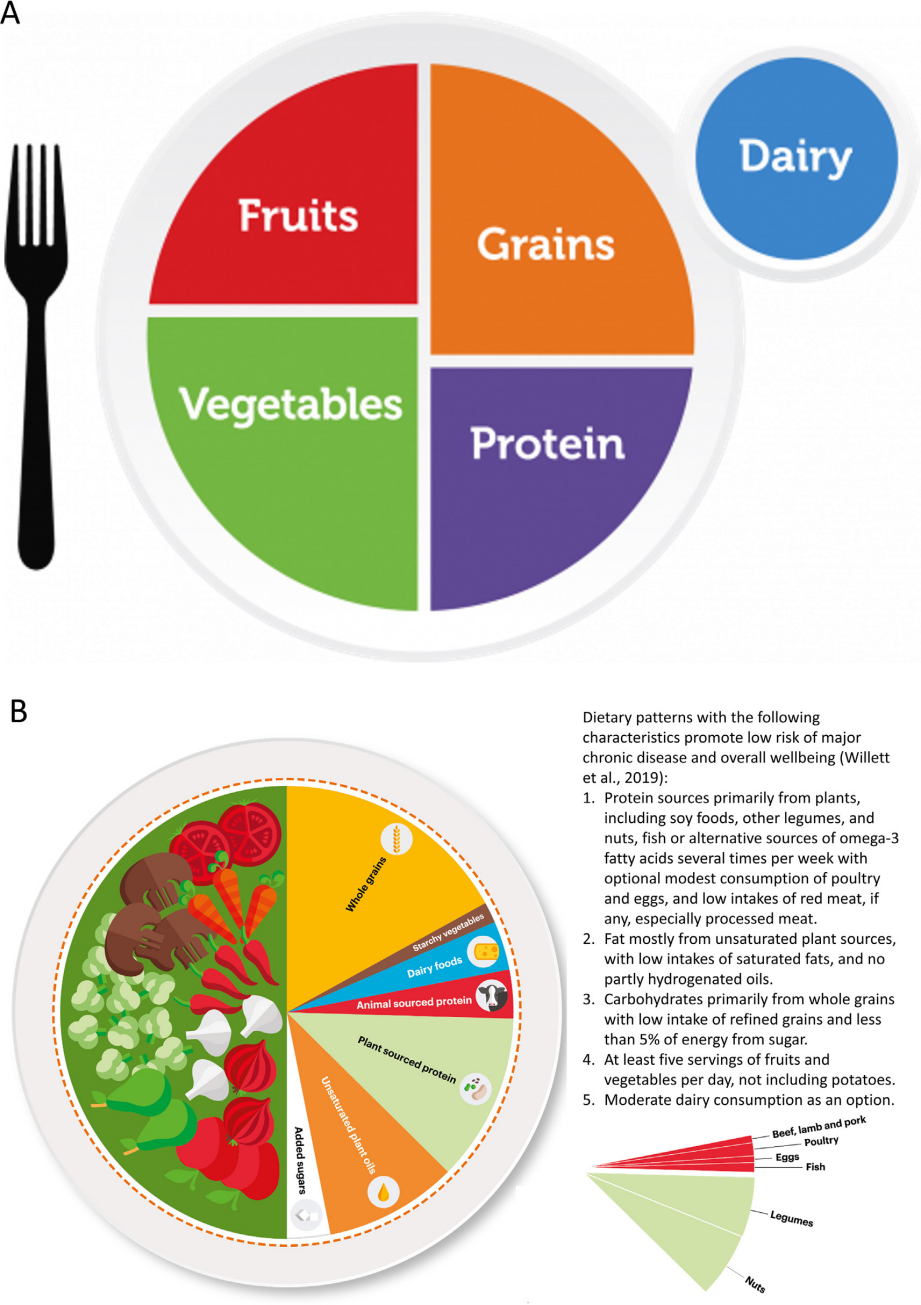
Dietary guidelines for the USDA, the EAT-Lancet study. Reproduced from reference (A) [18] and (B) [19] with permission.

The first starting point for consumers to adopt a healthy diet would be the guidelines provided by their governments and public health offices. An overview of these guidelines is given in [16] for 95 countries and by the FAO of the United Nations [17]. These guidelines advise the daily consumption of specific food groups, such as fruits, vegetables, proteins, grains, and dairy products. There are differences between countries, partly due to cultural and agricultural aspects determining the available and preferred ingredients. Examples of these guidelines are given in Figure 2 [18,19].

Even within a country, guidelines differ depending on the organization that composes them. For example, the USDA dietary guidelines recommend sourcing protein from “lean meats, poultry, and eggs; seafood; beans, peas, and lentils; and nuts, seeds, and soy products.” The American Cancer Society recommends “choosing protein foods such as fish, poultry, and beans more often than red meat, and for people who eat processed meat products to do so sparingly, if at all” [20,21]. The American Heart Association [22] recommends eating proteins from “mostly plants such as legumes and nuts; fish and seafood; low-fat or nonfat dairy; and, if you eat meat and poultry, ensuring it is lean and unprocessed.”

A large team of researchers, the EAT-Lancet Commission, recently composed dietary guidelines ([5], Figure 2) after evaluating the large body of knowledge. This is one of the most recent, comprehensive overviews to our knowledge. They analyzed multiple controlled feeding studies, long-term observational studies relating individual dietary components and overall dietary patterns to major disease endpoints and quality of life, and randomized clinical trials. The EAT-Lancet Commission advises that “healthy diets have an appropriate caloric intake and consist of a diversity of plant-based foods, low amounts of animal source foods, unsaturated rather than saturated fats, and small amounts of refined grains, highly processed foods, and added sugars” [5]. Other guidelines have been composed, for example, by Harvard University [23]. Note that these guidelines are sometimes disconnected from consumer preferences and attitudes, particularly for some age groups, and the required dietary guidelines do not necessarily fit with national eating habits and eating culture in a country. In addition, dietary guidelines also have an impact on the environmental impact of food supply. An example of a more comprehensive approach that considers these impacts is the SHARP diet, implying environmentally Sustainable, Healthy, Affordable, Reliable, and Preferred diets [24]. In addition, other factors, such as the financial challenges of households and the related cost of a healthy diet, also have an impact on the adoption of healthy dietary guidelines. Also, successful communication of these dietary guidelines is essential to ensure their adoption.

### Dietary choices

Dietary guidelines often focus on specific food groups (Figure 2). Even when one adheres to the dietary guidelines, the consumer has significant freedom to choose, for example, which types of protein or starch to eat. Particularly, the source of protein is a key determinant in distinguishing different diets, such as omnivore, semi-vegetarian, vegetarian, and vegan. Several studies have examined these diets’ health impacts, highlighting a few important findings.

The EAT-Lancet Commission [5] recommends consuming proteins primarily from plants, including soy foods, other legumes, and nuts. It is also suggested that fish or alternative sources of omega-3 fatty acids be included several times per week. Poultry and eggs can be consumed in moderation, whereas red meat, especially processed meat, should be consumed in low amounts or avoided altogether. They advise moderate dairy consumption as an option. In this context, a large prospective cohort study of 73,000 people in the United States and Canada was performed, called the Adventist Health Study [25]. The impact of 5 diets on mortality was analyzed. Compared with omnivores, the overall mortality risk was reduced by 9% for lacto-ovo-vegetarian/vegetarian, 8% for semi-vegetarian, 19% for pescatarian, and 15% for vegans. In a large prospective cohort study on 131,000 people in the United States, the impact of animal and plant protein on mortality was analyzed [26]. Overall mortality was reduced when replacing protein from animal sources with plant proteins, on average, 10% for every 3%-energy increment sourced from plant proteins. The analysis of data from a total of 166,039 women (data obtained from the Nurses’ Health Study and Nurses’ Health Study 2) and 43,259 men (data obtained from the Health Professionals Follow-Up Study) revealed that a higher intake of healthy plant foods is associated with substantially lower coronary artery disease (CAD) risk [27]. However, a plant-based diet emphasizing less healthy plant foods (such as fruit juices, refined grains, potatoes, sweets, and desserts) is associated with higher CAD risk [27]. The same group confirmed a substantially lower risk of developing type 2 diabetes with diets rich in plant foods based on data obtained from the same studies mentioned above, including a total of 160,188 women and 40,539 men [28]. Several studies also advocate the health effects of plant-based diets due to their observation of reduced mortality [6,29].

Other studies explored the impact of diet on healthcare costs. The Buddhist Tzu Chi Vegetarian study showed that changing the diet from omnivore to vegetarian reduced 15% of medical costs and 13% of outpatient visits for 2,166 vegetarians, leading to lower medical expenditure [30]. The Polypharma study on 326 patients aged 60 y and above showed that a vegan diet led to a 58% reduction in pills compared with a nonvegetarian diet [31].

Apart from the food group, the food processing level can also affect several foods’ health benefits. An example here is whole-food diets, where foods are consumed in their natural form after harvest, with no or minimal processing (for example, freezing, drying, or canning) and few added ingredients. In this context, the BROAD study tested a whole food plant-based diet in a 6-mo randomized controlled trial on 65 subjects [32], where subjects were advised to eat until satiation. They found that the whole food plant-based diet significantly reduced the BMI and cholesterol levels, which were the endpoints. It also had other health benefits for patients diagnosed with obesity, ischemic heart disease, or diabetes. Another study showed that 90% or more participants adhering to a whole food plant-based diet could improve LDL cholesterol, triglycerides, and systolic and diastolic blood pressure (BP) within recommended healthy targets [33]. These benefits could already be achieved in the short term (0.5–2 y) and do not necessarily need longer-term adaptation (5–10 y). Nevertheless, individuals can face hurdles in adopting whole foods in their diet for several reasons, including the availability of fresh, whole foods, their incorporation into cultural dishes, taste preferences, financial constraints and eating habits, social and family situations, and access to information on whole food diets and recipes.

In conclusion, dietary choices within a certain food group can affect health and influence the prevalence of diseases and health conditions. Replacing meat with plant-based foods and including whole, plant-based foods might be effective dietary strategies to support health. Access to these specific food groups is imperative here. Apart from financial access, logistical access should be enabled. This entails optimizing food production and SCs to reduce this bottleneck in supplying healthy foods to consumers or patients in need.

### Adherence to dietary recommendations

**FIGURE 3:**
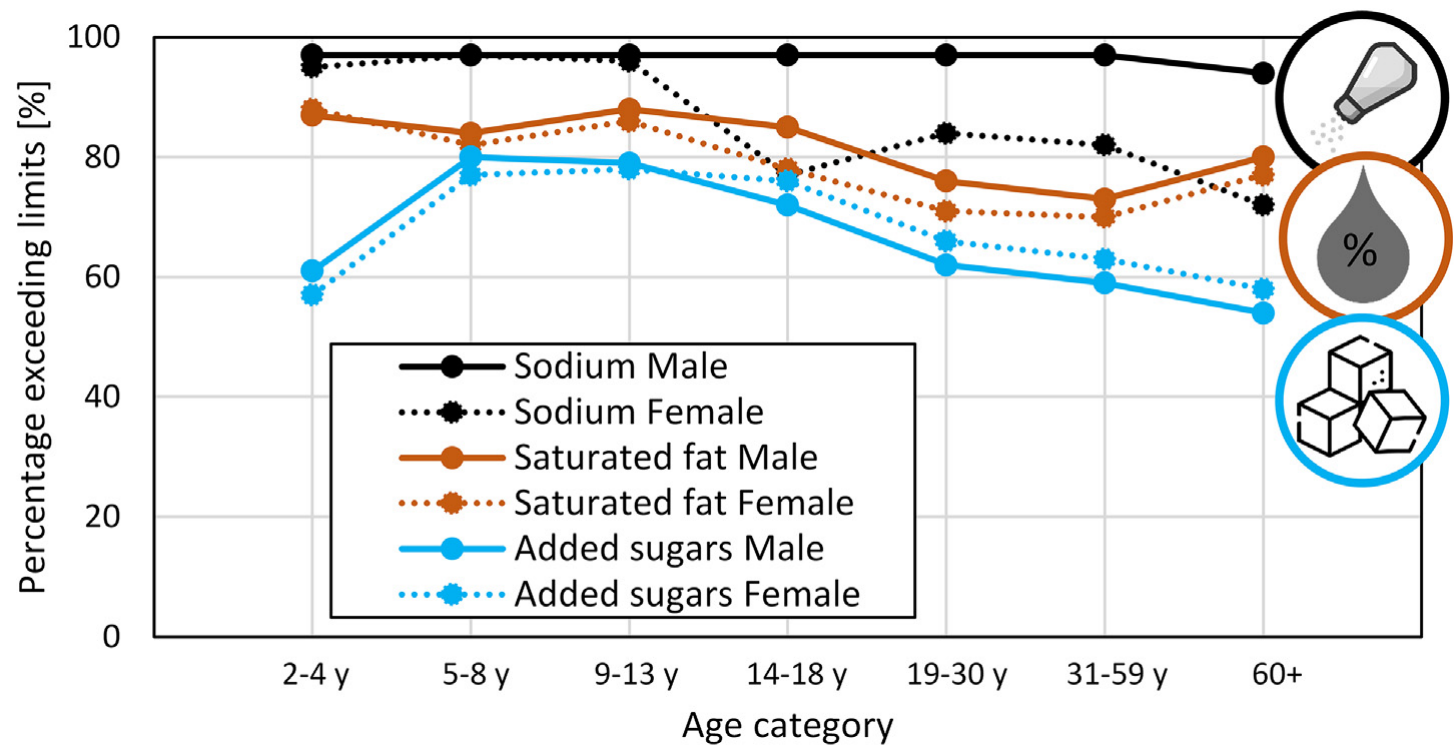
Percentage of people exceeding the average daily intake compared with the recommended range in the United States [37].

Despite the efforts to set up and promote these guidelines, many people still fail to comply with them [34]. In high-income and low- and middle-income countries, almost 40% of the population does not adhere to the national food-based dietary guidelines. Other studies even report a lower adherence, for example, only 10% adherence in the United Kingdom [35] or below 10% in Mexico [36]. Surprisingly, fruit and vegetables are adhered to relatively well in these studies. Animal protein consumption typically exceeds the recommendations. In some high-income countries, a high consumption of foods rich in added sugars, saturated fats, alcohol, and sodium was found. Data from the United States dietary guidelines [37] show the excess daily consumption of salt, saturated fats, and added sugars (Figure 3). Over 60% of the people do not meet the guidelines for almost their entire lives. Adherence to the dietary guidelines led to a lower mortality risk [35]. In short, consuming and adhering to a diet as prescribed in dietary guidelines increase lifespan.

## What is FAM?

### Definition

**FIGURE 4:**
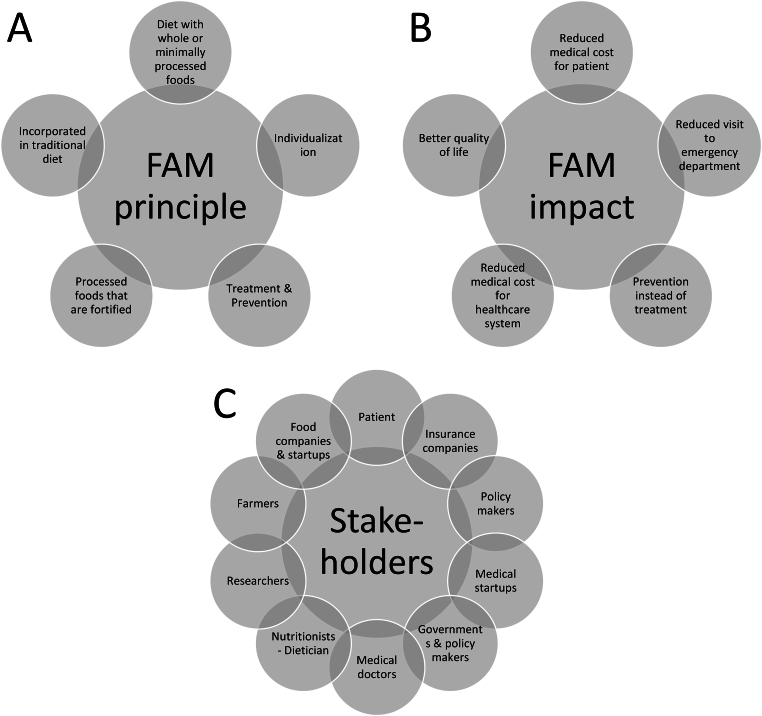
Overview of the (A) food-as-medicine (FAM) principle, (B) intended impact, and (C) stakeholders.

“Food-as-Medicine” or “Food-is-Medicine” is congruently used. FAM implies that food and diet are implemented as a part of an individual’s health plan, particularly focused on patients with a disease or a predisposition to specific diseases or health conditions. An overview of its principle, the intended impact, and the stakeholders is given in Figure 4. The aim is to *1*) promote good health and overall vitality, *2*) prevent and manage acute or chronic diseases or health conditions, *3*) reduce symptoms or even reverse the disease state, and *4*) support and complement conventional medical treatments. FAM is implemented through various dietary interventions and healthcare services that leverage the link between chronic or acute illness and nutrition.

In principle, the use of food for health has been around for centuries. Examples are Chinese, European, or Ayurvedic medicine. FAM is not just a rebranding of these concepts. Instead, FAM applies knowledge of the impact of food on health in combination with several recent technological advances to reach and treat patients in ways that were not possible before.

There are 2 main target groups for FAM interventions. First, we have populations or communities with high food security (with financial and logistical access to nutritious food) but who do not incorporate sufficient nutritious foods in their daily diet to prevent or treat diseases and health conditions. Second, we have populations and communities currently food insecure and do not receive the appropriate amount of essential nutrients in their daily diet. For both groups, an unbalanced diet can promote chronic and acute diseases and health conditions.

Food insecurity in a household can come from a lack of financial and logistical access to nutritious food due to the geographical location of the food not served by SCs. As such, healthy, nutritious food is not affordable or available by which socioeconomic drivers may negatively affect health [38]. Such situations are found in low-, middle-income, and high-income countries, such as food deserts and low-income communities. In the United States, cardiovascular disease, stroke, and diabetes disproportionately affect low-income communities [1,9,39].

### Principle

The underlying principle of FAM is that our food can significantly impact overall health and well-being. FAM implies adopting a balanced and nutrient-rich diet. Such a diet could include a combination of *1*) whole or minimally processed foods that provide required proteins, carbohydrates, fats, vitamins, minerals, fiber, and other beneficial compounds; *2*) functional foods, which can be foods such as some fruits, herbs, and spices with intrinsically present health-promoting compounds; *3*) processed foods that are fortified with functional compounds. We do not consider food supplements as a part of FAM, for example, vitamin supplements, because they are administered separately from a meal or food product. Apart from the measurable physiological impacts of FAM on health and disease management or prevention, there are also psychological benefits sought with FAM. Here, one leverages the social, emotional, and cultural impact of food and eating on mental health, mood, and stress. The principle, impact, and stakeholders of FAM are shown in Figure 4. These aspects are elaborated later in this work.

The concept of FAM induces a shift in conventional healthcare systems and medicine approaches. These rely mainly on managing diseases with pharmaceutical drugs and diagnosing health conditions with advanced medical technology. This approach is targeted to treatment rather than prevention. It has led to the development of many drugs to reduce or treat symptoms and the development of medical devices for diagnostics. FAM proposes dietary changes as a part of lifestyle changes, as a parallel line of prevention, and particularly supporting the treatment of health conditions. In contrast to dietary guidelines, FAM interventions have several target groups within a population.

### Hierarchical levels of FAM

FAM is implemented in several hierarchical levels for different target populations (Figure 1, adapted from [11,12]), often represented in a FAM pyramid. Interventions target entire populations down to interventions tailored to individual patient groups or patients with known (or diagnosed) diseases or health conditions. Target populations are food-insecure people and food-secure people who use food to prevent or treat health conditions. At the top of the pyramid (Figure 1), patients typically have a more severe health condition requiring more tailored and intensive treatment. Further down the pyramid, progressively larger groups of patients with less severe conditions can be treated through diet with more generalized interventions. The degree of individualization often determines the healthcare effort and costs. Several different categorizations are used. There is no consensus on whether government nutrition security programs and population-level or community-level healthy food programs should be considered part of FAM [40,41]. The following levels are typically present in FAM [11].

1) Medically tailored meals target patients with acute or chronic diseases or health conditions, such as cardiovascular or chronic hepatic diseases, nephrological disorders, diabetes, or obesity. Often, the patient is assigned to a treatment by a physician or a healthcare provider. A dietitian or registered nutritionist customizes the meal plan to the patient’s medical needs and health conditions. This FAM intervention is rapidly gaining traction [10], with several services and companies that tailor and/or deliver nutritionist-tailored meals to patients.
2) Medically tailored food packages or groceries target patients with less severe health conditions. A dietitian or registered nutritionist designs the partly prepared groceries, and patients pick them up and prepare the food themselves at home. After a medical diagnosis, these food packages are prescribed as part of a healthcare plan. This strategy targets people with health conditions or those lacking access to certain foods, such as fruits and vegetables.
3) Produce prescriptions have a similar target group as medically tailored food packages, but the patient needs to put more effort into preparing the meal. Produce prescriptions are simple to implement. Patients can incorporate also the ingredients in their local cuisine, which can ensure longer-term adaptation of the diet. A typical example of produce prescriptions would be farm-produce pickup as a part of community-supported agriculture. Nutritious food referrals are similar to produce prescriptions. Nutritious food referrals provide funds for free or discounted nutritious foods. Individuals must receive referrals or plans from healthcare providers after being identified as having a diet-related disease or at risk of developing it.
4) Government nutrition security programs (Supplemental Nutrition Assistance Program, Women, Infants and Children, Healthy Incentives Pilot, emergency food programs) target large groups of people. Despite these government food assistance programs, eligible patients are often not reached because they are not known or not aware. Identification of these patients and information dissemination within are essential for their success. Healthcare systems could actively assist here.
5) Population-level or community-level healthy food programs stimulate easy access to affordable, nutritious foods for communities at risk of health conditions or diseases. These programs stimulate interventions to reach nutrition security and health equity that account for sociocultural and geographical boundary conditions limiting food security. Such food programs can target, for example, the mitigation of food deserts.

An overview of recent studies and outcomes on medically tailored meals, medically tailored food packages, and nutritious food referrals is given in [41]. Most of the research is done on nutritious food referrals or produce prescriptions. Several studies showed that produce prescriptions or nutritious food referrals could significantly increase the intake of fresh fruit and vegetables [41], leading to associated health benefits. Produce prescriptions also improve diet quality [42] and reduce the BMI [43]. For example, it lowered BP and the BMI for 3,881 individuals after 6 mo [44]. Medically tailored meals have been shown, for example, to reduce patient admissions by 49% and healthcare costs by 16% in a retrospective cohort study of 1,020 participants [45]. A recent report investigated the impact of national implementation in the United States of medically tailored meals in Medicare, Medicaid, and private insurance for patients with a diet-related condition and a limitation in activities that are essential during daily living [13]. Medically tailored meals are estimated to avoid 1.6 million hospitalizations and save 13.6 billion USD in healthcare costs. The same report identified that produce prescription programs could avoid 292,000 cardiovascular events and save 39.6 billion USD in healthcare costs.

Apart from the perceived health benefits, which are only demonstrated after adopting the diet for a certain time, a financial incentive is often essential for patients to start adopting the prescription first. A simulation study on 82 million adults showed that subsidizing fruit and vegetables by 30% could prevent 1.93 million cardiovascular disease events, gain 4.64 million quality-adjusted life years, and save $39.7 billion in healthcare costs [46]. This study assumed that individuals would be funded through the nation’s (United States) 2 largest public insurance programs: Medicare and Medicaid.

This FAM pyramid does not include holistic coaching of individuals who tailor the diet to prevent and treat acute or chronic diseases or health conditions. These services range from individuals visiting a dietitian to high-end, more costly services for athletes, heads of state, or other top performers. An example of a company offering holistic coaching, including nutrition, is Hintsa [47].

Although we did not consider them in this study part of the FAM pyramid, meal kit companies can also play an important role in stimulating people to cook themselves and learn new recipes instead of ordering takeout. In that way, individuals have better control over their eating, which could lead to healthier eating habits. It is essential that the recipes and ingredients promote health. Note, however, that the incentive of these companies is not necessarily to provide healthy food in the first place but to provide appealing, tasty meals to maximize customer satisfaction. Even though these companies are business driven, they provide a gentle way to educate populations to cook and eat differently. The service is, however, often deemed to be expensive and not subsidized, making it still a niche market. Examples of meal kit companies are HelloFresh, Blue Apron, Home Chef, and Sunbasket. Such meal kit companies, to some extent, contribute to the culinary training and education of the population. Culinary education is considered essential for the healthy and nutritious eating habits of the wider population. This involves ingredient sourcing, the preparation of ingredients, cooking techniques, food safety, reducing food waste, and food storage.

### FAM compared with precision nutrition and personalized nutrition

FAM should not be confused with precision nutrition or personalized nutrition as there are subtle differences [48].

FAM relies on foods to prevent diseases and health conditions and support treating them, complementary to conventional medicine. The focus here is on nutrient-rich, whole foods and minimally processed foods incorporated into traditional diets. FAM is not focused primarily on individualized dietary recommendations but rather targets larger patient groups at different hierarchies and specific diseases (Figure 1).

Personalized nutrition aims to tailor a diet and its nutritional composition to an individual [49]. This implies accounting to some extent for the patient’s unique genetics, metabolism, microbiome composition, lifestyle choices, living environment, cultural dietary habits, and personal preferences. Individualized dietary recommendations are based on lifestyle, dietary habits, and health status assessments. The diet can also include food supplements and functionalized foods. To this end, blood tests, genetic tests, and metabolic profiling are used and combined with advanced data analytics. Personalized nutrition tailors the diet to 1 individual, using advanced technologies to promote health but not necessarily only to help treat disease.

Precision nutrition is often used interchangeably with personalized nutrition [50]. The subtle difference would be that precision nutrition relies heavily on advanced technologies to tailor the diet to the patient, such as genetic testing, metabolomics, microbiome analysis, bioinformatics, and the associated data analytics. This approach is more science and biological data-driven and accounts less for the patient’s lifestyle choices, living environment, cultural dietary habits, and personal preferences.

## Recent Advances in FAM

FAM has received increasing attention on several fronts and stakeholders in the past decade.

Multiple foundations and other governmental and nongovernmental organizations have recently promoted FAM. An example is the Food Is Medicine Coalition [10], established recently as an association of nonprofit medically tailored food and nutrition service providers. They focus on medically tailored meals for people with acute or chronic illnesses. Food is Medicine Massachusetts [12] is a multistakeholder coalition with over 80 organizations participating with diverse expertise across the food and healthcare systems. They work on FAM services at every part of the pyramid and map all available services in the Massachusetts Service Inventory map [51]. The Rockefeller Foundation supports initiatives to build the FAM evidence base, advocate policies that support healthy food, and remove barriers to reaching more diverse populations that are currently disadvantaged [1,9]. They plan to mobilize 250 million USD to build a national Food is Medicine Research Initiative with the American Heart Association [52].

The food and nutrition industry, particularly startups, is increasingly active in this field and attracting significant funding [53]. This movement seems to follow the previous wave of technology startups focusing on previous food technology innovations. Examples are algae, fungi, and pea proteins for manufacturing plant-based meat alternatives, cell-based meat, or insect proteins [54]. After these hypes, FAM, focusing on further individualizing food and related auxiliary enabling technologies, is receiving attention now. We give some examples that have been identified by the authors during their years of work in this field:

1) Instacart Health aims to provide individualized groceries for patients leaving the hospital, to support recovery, or to have virtual food pharmacies for doctors to prescribe food in the same way as medications.
2) Uber Health aims to enable physicians to prescribe medically tailored meals or groceries to patients. This adds to Uber’s healthcare services, such as offering patient transportation on healthcare plans or medication delivery.
3) Noom provides a digital platform through which users can improve personal health and well-being. They combine psychology, technology, and human coaching to empower people to improve their health. They focused at first on weight management but now expand chronic and nonchronic conditions, such as stress and anxiety, hypertension, and diabetes. They assist individuals in choosing nutritious foods and in meal planning.
4) Brightseed aims to discover plant-based bioactive compounds or ingredients that can be used to improve health. They use AI-based technology for such discovery and now focus on compounds affecting gut barrier function, liver function, and metabolic processes.
5) Season Health is a FAM platform targeted to people with health conditions, such as diabetes, high BP, weight, heart disease, chronic kidney disease, celiac disease, or high cholesterol. In a one-on-one with a dietitian, they build an individualized nutrition plan. They prescribe food, enable you to order groceries or meals, and do periodic follow-ups with the dietitian.
6) Second Nature provides an app focused on weight loss through a fitness program with nutritional plans.
7) Zoe provides an individualized nutrition program based on at-home tests measuring blood fat, blood sugar, and gut microbiome health. On the basis of your profile, they composed food combinations to boost your health.
8) InsideTracker uses data on the patient in order to provide personalized recommendations to their customers. They rely on data on the physiological and demographic profile, blood tests, DNA, nutrition, lifestyle, exercise, and information from wearable devices [55]. These biometric and other data are used in a data-science algorithm to provide personalized recommendations that are communicated through a customer interface platform.

These startups strongly focus on using digital apps and platforms for tracking diets, nutrition information, individualized recommendations, and data-driven meal planning. These startups also heavily rely on data science and AI. Most startups mainly work in the upper part of the FAM pyramid because the lower part is dealt with more by dietary guidelines, governments, or government-funded projects (Figure 4). Some are even leaning toward personalized and precision nutrition (FAM compared with precision nutrition and personalized nutrition section). Governments also support mobile app development efforts [56], but these initiatives are much more scarce than those of startups.

Health insurers also increasingly promote healthy lifestyle choices with their customer-facing web- and mobile-based services [57]. Similar to governmental initiatives, their focus is generally on the lower part of the FAM pyramid, that is, increasing population health by providing prevention services and health literacy content, often in combination with reward programs, concerning diet and nutrition. Examples are active365, Helsana Coach, or BENEVITA by the Swiss health insurers CSS, Helsana, and SWICA.

In conclusion, we see momentum in research and industry, particularly by startups, driving individualization of FAM. This means the diet is tailored to the patient for prevention and treatment. The activities in medically tailored meals, food packages, or produce prescriptions target preventive interventions that reach many more people simultaneously instead of only a small number of patients. Reaching so many people simultaneously is enabled by digital innovations and connectivity. This implies that we are stepping away from the FAM pyramid structure and are trying to reach each individual in a population by providing expert dietary coaching. These activities, therefore, reach toward personalized nutrition or even precision nutrition, but now with a specific focus on supporting the treatment of the disease, in addition to prevention, and relying on traditional diets and largely on whole or minimally processed foods.

Such development and solution offering to more people have recently become possible, scalable, and commercially viable for companies. Drivers are technological developments that enable gathering detailed information on multiple aspects of each individual’s body and lifestyle. Examples are as follows:

1) Digital behavior change interventions gather data about vulnerable states of individuals on a large scale to deliver individualized meal plans and information to take action and motivate users [58].
2) Genomic and microbiome testing kits to map the patient’s health profile, often at home.
3) Real-time monitoring of the patient through wearable technology of several body functions [59].
4) Data analytics and data science to leverage data on a large number of patients.
5) AI-driven predictive and prescriptive analytics to prescribe nutrition and automate dietary advice, supporting dietitians ([55]).
6) Telemedicine enables direct worldwide access to dietitians via digital health platforms [58].

## Impact of FAM and Dietary Guidelines on Primary Food Production and Food Technology

Dietary changes will affect the food we produce and process, including those induced by adherence to general dietary guidelines or FAM interventions. The resulting demand pull from the consumer side will be mainly for interventions at the lower part of the FAM pyramid (Figure 4), as they often involve larger populations simultaneously. FAM interventions higher up on the pyramid, such as medically tailored food packages, serve a more niche customer base so that they will have a smaller impact on the food SC. Such a FAM-driven consumer pull can induce a higher consumption of particular foods, such as fruits and vegetables, whole grains, or plant-sourced proteins. This will impact the entire SC, including production volumes, transport, and storage, which are often refrigerated. We discuss these envisaged changes mainly from the perspective of fruit and vegetables because these make up a large part of the recommended diet (Figure 2).

An example of how changes in consumer health perception of some food products, together with the globalization of the market, led to a huge impact on production volumes is seen for avocado consumption in the United States. Here, consumption rose from 716,353 tons worldwide in 1961 to 4,066,610 tons in 2011 and 8,685,672 tons in 2021, driven by the promotion of this food for its health benefits [60]. This is a 10-fold increase in 60 y and has even doubled the amount produced in the past 10 y. The global population increased from 3.1 billion people in 1961 to 7.9 billion in 2021 [60], which is only a 2.6-fold increase. Similarly, the perception of the health benefits of lemons has significantly increased demand over the past years, for example, during the COVID-19 pandemic. Consumption has risen from 2,619,753 tons worldwide in 1961 to 15,046,903 tons in 2011 and 20,828,739 tons in 2021 [60].

However, the impact of FAM-induced changes on the food SC is difficult to predict and largely depends on consumer trends, marketing, and social media. FAM interventions will likely have a long-term impact on food production and processing.

### Food production changes and impacts

#### Changes

FAM interventions and—more generally—the transition to more healthy and sustainable diets could induce several changes in the food SC, particularly in food production. The appropriate amount of food for a healthy diet is not produced globally. Our current agricultural production is not tailored to match our government’s dietary guidelines, which are essential to prevent diseases in a FAM context. For example, we do not produce sufficient fruit and vegetables to meet these nutritional guidelines for the world population [61,63]. So, even if we want to adopt a healthy diet, not everyone can due to a shortage. However, serving selected patients in a population with FAM interventions might be more feasible.

For FAM and more general, changes toward a more healthy and sustainable diet would imply that we would need to act toward solving these bottlenecks:

1) Produce a larger volume of the appropriate foods to fill the production gap and supply nutritious and sustainable foods.
2) Reduce losses of food and its nutrients throughout the food system, as losses are currently huge, especially for fruit and vegetables. Over 20% of fresh produce is lost, on average, worldwide from harvest to distribution [62].
3) Adjust prices of these foods to make them more affordable, and change incentives so people find these foods more attractive, for example, through subsidies. FAM produce prescriptions or government subsidies have been shown to significantly increase the intake of, for example, fruit and vegetables [41,63].
4) Reduce demand and production of less healthy, less sustainably produced foods and the foods we already produce in sufficient amounts nationally and globally, such as cereals and starches. This reduction could partially occur automatically: if we eat more healthy foods and eat until satiety, we will eat less of the other foods. This is strongly dependent on the behavior change and discipline of the consumers.

#### Impacts and challenges

##### Accessibility

Even if we produce healthy food in certain agricultural regions, there will still be many hungry and malnourished people due to lack of access to food. The geographical, financial, and social accessibility in some regions remains a bottleneck. Geographical accessibility to healthy foods is problematic in remote areas and food deserts in cities. Food deserts are actually “healthy-food deserts” and at the same time “fast-food heavens.” The geographical accessibility is also linked to socioeconomic factors, as these areas are often populated by low-income communities. Stimulating financial accessibility to healthy food is challenging, but governments should strive to achieve this.

##### SC and infrastructure

We must adapt SCs and infrastructure if we produce other foods, such as more fruits and vegetables. An incentivized market push to produce more healthy food via a consumer pull or governmental interventions would imply that the capacity of all stakeholders (growers, pack houses, transport, and shipping companies, importers, and retail storage space) should increase. This requires preharvest investments in machinery, fertilizer, and agrochemical compounds.

After harvest, cereal grains or pulses are low-moisture content foods that typically do not need refrigeration but an appropriate hygrothermal climate and packaging, so a “dry chain” would be appropriate [64]. In contrast, perishable produce requires a cold chain after harvest. This implies more refrigerated storage space, trailers, and containers. Investments in cold chain infrastructure will be required, with an associated environmental impact. Fresh produce also contains much water, and their nutritional density per kg of food is not that high, requiring a lot of storage space and transportation costs to move these foods. These products are also sourced in different agricultural regions with different (marine) accessibility and trade routes.

**FIGURE 5:**
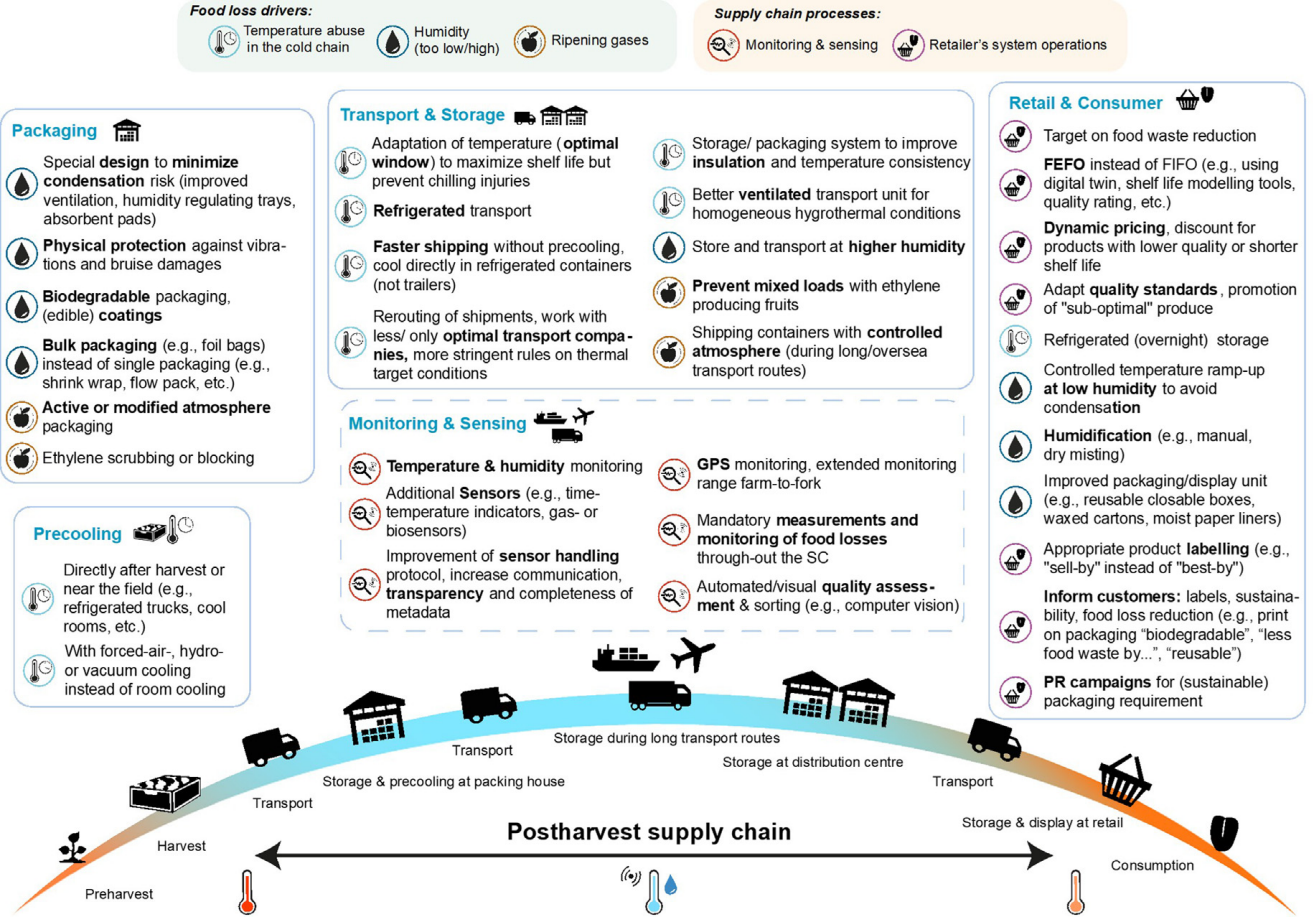
Food loss and waste-reducing strategies were mapped for different drivers and grouped for several stages along the postharvest supply chain of fresh produce. SC, supply chain; GPS, global positioning system; FEFO, "first-expired-first-out"; FIFO, "first-in-first-out"; PR, public relations (reproduced from reference [65] with permission).

In addition to cooling, several other postharvest technologies can be applied to optimize SCs, of which an overview is given in Figure 5 for fresh fruits and vegetables [65]. Innovations and research are required in these fields to preserve nutritional content and food quality better and reduce food waste. FAM interventions, in particular, could also require more product segmentation, in contrast to bulk food supply, as more specialized and individualized products or meal packages need to be composed.

##### Seasonality

Several fruits and vegetables are unavailable year round and produced only in their growing season. Some fruits, such as apples and pears, can be stored in a controlled atmosphere for months, but most fruits and vegetables have a more limited storage life. These foods form an important component of FAM interventions. A few options exist for patients and consumers to access a constant yearly healthy food supply at guaranteed volumes. One could adopt eating seasonal and locally produced fruits and vegetables. This can pose challenges for consumers as some food products are preferred over others. The limited availability of these foods in some seasons can hinder the adoption of FAM interventions. This can be mitigated partially by growing cultivars that are more climate- and weather-resilient than others, resistant to microbiological attacks, or have a longer storage life. Such storability and marketability advantages made some cultivars dominate the industry, for example, the “Hass” avocado or the “Conference” pear in the EU and the “Bartlett” pear in the United States. A second option is to provide patients with a year-round availability of certain food products, by extending and optimizing international import SCs.

##### Functional foods

Some foods have very high concentrations of compounds known for their health-promoting properties or anti-inflammatory or analgesic effects, although further scientific substantiation of their health effects is needed. Often, such foods are called functional foods or superfoods when consumed as a whole. An overview is given in [66,67]. Examples are pomegranate, several berries, flaxseed, chia seeds, turmeric, ginger, kale, spirulina, broccoli, oats, or baobab. The production of these foods could be stimulated, but some are less straightforward to obtain worldwide. The reasons are that they are costly and delicate to grow. Others require a dedicated postharvest SC or processing facilities to transport and preserve them. The environmental impact of such foods is often significant. Therefore, it might make sense to source functional foods regionally or nationally.

### Food processing changes and challenges

#### Changes

Several FAM interventions, for example, medically tailored meals and produce prescriptions, incorporate whole foods, such as whole grains or fruit and vegetables, and minimally processed foods that are dried, frozen, or canned. Several of these foods are then further processed at home, for example, by cooking into a meal. In addition, FAM interventions can incorporate more processed foods, for example, plant-based meat substitutes or foods enriched with functional compounds, such as vitamins. Such food processing can provide an essential way to trigger consumers to eat certain foods and nutrients that support their health and add nutritional value to existing ingredients. Respecting consumer preferences and eating habits in FAM interventions is essential for successfully adapting the meals. As such, preprocessing the foods in meal packages, for example, to change texture, structure, or taste, can aid in increasing consumer adaptation and administering the necessary food components. FAM interventions and dietary guidelines will, therefore, require a shift to making whole foods, minimally processed foods, and selected processed foods more available and appealing.

#### Impacts and challenges

Processing is done because of the benefits it induces. Thermal processing, for example, helps to prolong shelf life, inactivate food-borne pathogens, improve digestibility and bioavailability of nutrients, and improve palatability and sensory aspects [68]. However, thermal processing, including cooking at home, can also reduce the nutritional content of the raw products [68,69]. For a FAM context, it is essential that the advantages of processing target improving the patient’s health and that the possible negative impacts of processing are also acknowledged and managed.

Apart from promoting technologies that support FAM interventions, we see a possible increase in the technologies that enable FAM, such as the centralized production of healthy ready-to-eat meals and delivery logistics of groceries or meals. This will require the development of auxiliary technology, such as mobile cooking and dietary-coaching apps, delivery packaging and containers, or even drone-based deliveries or other delivery robots. In addition, we expect that home-cooking equipment, such as cooking robots for home consumption, will play a more central role. Also, cloud kitchens or virtual restaurants are expected to grow. These food services enable only delivery and pickup based on online or phone ordering.

## Which Factors Influence the Effectiveness of FAM?

**FIGURE 6:**
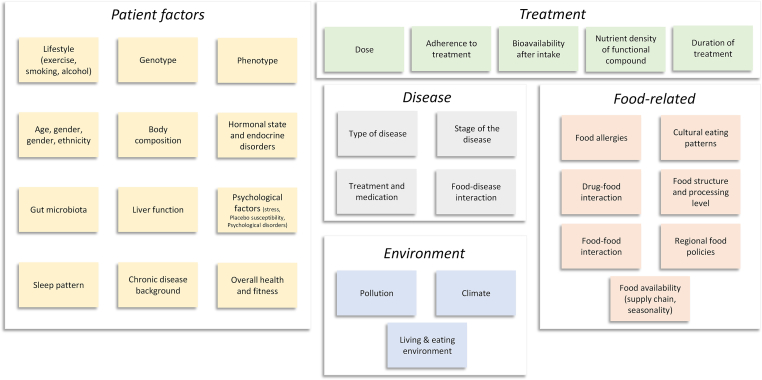
Selected factors impacting the effectiveness of a food-as-medicine treatment.

When prescribing a diet along any of the FAM pyramids to patients (Figure 4), the targeted effectiveness of the treatment will depend on several factors. These include, among others, the patient’s overall health state, diet, nutrition availability, food cost, environmental impacts, and the patient’s adherence to the treatment, as well as all factors that impact such adherence. We listed the factors that influence a FAM diet in Figure 6, particularly those that affect the uptake of nutritional compounds. These factors all contribute to the extent to which food compounds can be used for medical and health-promoting purposes.

Often, the desired effect of nutritional compounds with a targeted health or medical impact cannot be reached for several reasons. One example is the bioavailability of the targeted compound. Bioavailability reflects the component’s potential to reach the targeted site of action in the body by entering the systemic circulation and getting absorbed. In other words, it means how much of the compounds reach the target site to have an impact. When we administer a certain food for its health or medicinal properties, we target a certain nutrient density and bioavailability of the food to estimate the daily dose.

Adherence to FAM interventions is another important aspect that will affect their effectiveness. Adherence has several dimensions and challenges. First, the patient could be financially incentivized to start and adhere to selected FAM interventions and programs (for example, produce prescriptions). However, it is essential that this financial support continues for a sufficiently long time to guarantee compliance. An additional challenge lies in the dietary habits and cultural food preferences, which can conflict with the FAM intervention. In addition, lifestyle aspects, such as work schedules and lacking cooking skills, can hinder the adoption and consistent adherence to FAM interventions. In addition, adherence is strongly linked to the inherent motivation of the patient and the self-observed health impacts. Therefore, clear, immediate effects of dietary changes (measured or self-perceived) would likely help to increase adherence. It should be avoided that these effects are less noticeable as with the alternative, namely medication, as this can induce patient frustration and reduce compliance. Finally, it is important to identify the required time the treatment needs to be adhered to, namely, whether short or long-term, to be effective. Short-term FAM interventions could help to avert acute health issues, but often, long-term healthy diets should be targeted.

As another example, food–drug interactions must also be accounted for when a medical treatment is given. Some foods can inhibit the effect of certain medications, for example, fruit juices or high-fat foods [70,71]. However, food or food components can also be a strategy to prevent drug-related side effects, for example, in antibiotic-associated diarrhea [72,73]. Therefore, taking drugs and food or meals in the proper combination or separately is important.

The food structure after minimal processing (for example, freezing, drying, or canning) or more extensive processing can also affect nutrition uptake, including the bioavailability of the targeted compounds. For example, an orange fruit can be eaten as a whole or as fruit juice. However, the structure of our food is often prescribed by how consumers want to appreciate and experience the food and meals they eat. Food structure is also used to transform foods to create more appealing products and to make healthier food compounds, such as fruit, more attractive to consumers. Examples are “apple chips,” “fruit sticks/bars,” or fruit-cereal bars. Food structure (softness, texture, color, and flavor) is determined by the processing applied in the factory or at home and subsequent chewing of the food. Food structure is, therefore, also essential to consider in effective FAM interventions. As an example, the amount to which we chew our food affects appetite, food intake, and hormone release [74]. As a result, it also affects our nutritional intake and weight [75]. Chewing difficulties can lead to nutrition deficiencies, partly due to reduced uptake. Another reason is that several nutritious foods that are more difficult to chew, such as raw fruit and vegetables, are left aside [76]. As another example, it seems that eating structurally intact foods can also positively affect weight. For fruit and vegetables, for example, some nutrients can remain trapped within the plant cells during digestion [7]. Although we indeed excrete valuable nutrients, the glycemic response after eating the food will be reduced, for example, compared with drinking fruit juice or a smoothie.

## Are We Going in the Right Direction with Individualized FAM?

Recent trends point toward more segmentation and individualization of the diet in FAM. Segmentation here implies a step in between interventions for large groups of patients and complete individualization. Segmentation focuses on specific target groups of patients without providing specialized treatment for each individual.

There is no one-size-fits-all approach to each medical treatment or preventive health action. Therefore, the vision is to provide tailored dietary advice and the associated food to each of us at a reasonable cost. This shift implies that FAM ventures in personalized nutrition, with a clear medical focus on treating specific diseases and serving larger patient groups or segments. A question remains on how far we can and should go in tailoring the diet. In this section, we explore *1*) how we can meet our target with the tailored advice we are providing, *2*) that an essential but missing next step is to prove that segmentation and individualization work on a larger scale, *3*) why we seem to be skipping this step.

### How can we meet the target?

Designing an effective individualized diet is a challenging task, given the many genetic and environmental factors that affect the efficacy of the diet (Figure 6). There are many ways in which the diet cannot reach its envisaged effect. It will be challenging to tailor the administration of compounds through food to patients, even for a nutritionist, and likely also when having advanced (future) technologies at our disposal.

Similar problems are currently appearing in personalized medicine. Here, delivering 1 single drug or compound in a way tailored to a single patient is already challenging (for example, [[77], [78], [79], [80]]). The complex interactions of the different factors (Figure 6) make it immensely time-consuming–thus costly–to elucidate accurately an individualized therapy for a certain disease. Tailoring a complete diet with the entire nutritional profile for a single person would need to be done for several months, as this timeframe is typically required to be effective. Often, the models that are established to elucidate such nutritional profiling for a single person would require large datasets on many people. In addition, the interaction of FAM interventions with the used medication also needs to be considered.

Such efforts are successfully implemented for top athletes, heads of state, or other government officials. Here, advanced efforts are made to personalize the diet for the prevention and treatment of health conditions and to boost performance. Entire teams of nutritionists, physicians, and exercise coaches are occupied with measuring and continuously monitoring these individuals using the newest technologies in monitoring and medical diagnostics. This creates a clear technology push and successfully improves performance. However, given the high amount of resources required, the roll-out to a larger number of individuals is currently not scalable or financially viable, so more empirical approaches are often applied.

The collection of large data sets, such as intake, activity, and omics data, is not the main challenge in personalized nutrition. The challenge is translating the collected data to science-based personal advice encouraging patients to make necessary diet and lifestyle changes. For many food groups, functional foods and specific diets, nutrition, and health relationships have not yet been fully established.

Nevertheless, several startups are targeting to provide individualized diets or dietary advice to the masses (FAM compared with precision nutrition and personalized nutrition section), work with available data sets, and try to bypass the large number of resources currently spent for effective individualized treatment, such as athletes. They target a more granular level of individualization, so patient typologies, and may have less success for each individual. However, they need to prove how effective their solutions are in the long term and which degree of individualization they can reach.

### The essential next step is proving individualization works on a larger scale and determining which level of individualization we need

We still do not have sufficient proof that individualized FAM strategies currently offered (FAM compared with precision nutrition and personalized nutrition section) can be generalized to the masses. We, therefore, cannot say if these more pragmatic strategies that avoid the huge resources currently needed are effective. One reason is that this field, with its commercial players, is rather new. Clinical trials with robust study designs must prove that the proposed individualized treatment, for example, dietitian-assisted mobile applications, significantly improves individuals’ health. As the target is to improve each individual’s health, not the population as a whole, success should be measured by the improvements for each individual separately. The nutritional advice may miss some key aspects of the patient. This can make the patient experience some health benefits, but the promised added value of the individualization is not reached. Researchers are still advocating for more clinical studies to test the impact of using FAM to treat a specific disease [14,81]. There is a low number of clinical studies on several FAM interventions, given the recent trend. This entails that longer term studies of FAM interventions are also essential in the future. Especially long-term adherence to FAM interventions will need to be investigated and monitored to show how sustainable and well received the interventions are.

We also do not know to which level of granularity the individualization is effective when scaled up to a larger population, so the costs justify the health impacts. These levels of granularity are, for example, the individual, a certain age group, an ethnic group, a fitness level, a gender group, or a patient or disease group. We need studies to assess if the costs justify the health impacts of individualizing diets for FAM.

Here, it is essential to prove that by providing a tailored diet for each individual, we get a statistically significant gain in health benefits with the help of advanced technologies (FAM compared with precision nutrition and personalized nutrition section). We need to compare here, as a baseline, to a person adhering to general dietary recommendations. Several previous studies have found significant benefits by prescribing a “general” dietary change or adopting 1 or more foods.

Several aspects need to be covered when trying to prove this added value. First, randomized controlled trial studies would be required based on patients’ preselected biological characteristics to compare the health impacts of a general recommendation for a dietary change—for example, from omnivore to a whole food plant-based diet—to an individualized diet. It is also worthwhile to consider other designs in the context of personalized nutrition, such as *n*-of-1 trials and aggregation of numerous *n*-of-1 trials. Second, the costs of both treatments should be compared with the reduction in healthcare costs, similar to what has been done for other diets [30,31]. Third, treatment adherence must be evaluated over a longer term, which is also key to successful health impacts. Combining these elements can increase trust that individualizing treatment for individuals is better for human health and healthcare costs compared with more coarse-grained dietary changes.

### Why are we skipping a step, and might we be going in the wrong direction?

Many companies and startups provide individually tailored nutritional advice, mainly through digital-assisted solutions, such as mobile applications. Why are they betting on individualizing FAM even though the long-term added benefits of such an approach over general dietary advice are not always sufficiently proven yet?

There are several factors that could be playing a role here. First, a wider customer base means a better business case. Companies will depend more on customer adherence to secure growth than when serving a few athletes or wealthy customers. Second, individualized dietary advice appeals to people and forms part of the “Quantified Self” movement. Here, people deploy self-monitoring, including nutrition and health, using technologies such as wearable smart devices. Having their own nutritional/dietary fingerprint can stimulate affirming their identity, but it can also be helpful in disease prevention as it enables people to adopt lifestyle changes based on self-monitoring in a timely manner. Third, such individualization is enabled by several existing technologies, which people already use daily and are familiar with. Examples are wearable devices currently used to track one’s general health and movement and mobile apps, which provide dietary information, meal planning, and meal ordering. These technologies are essential to successfully implementing and marketing new products in the food industry. Fourth, the market for individualized FAM was not large until recently, so there are not a lot of established competitors. Fifth, there is a high chance that the customer will feel a noticeable health effect. One reason is that there are already proven health effects from adhering to dietary recommendations. If the service provided would already stimulate that, it will likely help improve the health and well-being of the customer. In short, there is a customer base that is largely untapped and open to the FAM concept and services, which might lead to a fast move in the direction of fully individualized FAM.

## Conclusions and Outlook

We analyzed FAM interventions from the viewpoint of their impact on food SCs, food production, and food processing. Using “FAM” has a high potential to reduce healthcare costs due to its preventative approach and by complementing medical treatments, reducing the amount of medications taken. Furthermore, this might lead to improved disease management, reduced disease severity, and fewer inpatient hospitalizations. FAM interventions will impact the foods we grow, the products we produce from them, and the R&D and technologies we will develop to produce them. This trend could create a demand for certain food products (for example, fruit and vegetables) and demand–pull on technology development. At the same time, a technology push would enable new opportunities to better serve patients in need with FAM interventions. We state the following findings:

1) FAM is enabled by several technological advances that allow patients to be reached and treated in new ways that were not feasible before.
2) Dietary interventions, even adhering to the country’s dietary guidelines, effectively help to prevent and treat diseases and acute and chronic health conditions such as cardiovascular disease, different types of cancer, type 2 diabetes, or obesity.
3) Better implementation and promotion of dietary prescriptions by physicians and healthcare insurance are critical, next to pharmaceutical prescriptions, to increase acceptance by patients and incentivize them. FAM can thereby complement conventional medical therapy.
4) FAM interventions have many different levels of implementation, which are depicted in the FAM pyramid. Financial incentives and support greatly impact the adaptation of these interventions.
5) Companies and startups are betting big on the individualization of the diet. They provide different digital-assisted dietary advice, coaching, or meal delivery. This approach often simplifies tailored dietary advice to be able to scale for the masses but still needs more rigorous clinical validation. True individualization is less likely to be achieved without a systems approach that accounts for the patient’s genetics, lifestyle, and environment and its response to all nutritional components inside the many food items consumed. An issue is that the targeted effect—the individualized nutrition effect—is not reached fully. However, improving the patient’s health and well-being can be likely achieved by improving the current diet, as many people still do not adhere to the standard dietary recommendations.
6) Essential for the FAM hype is to identify the improvement that can be achieved by the individualized approach, compared with a more general adaptation of a healthy diet. As long as this is not shown, it will not be proven to the customer if they get value for the additional tailored services they rely on.
7) FAM interventions will require a shift to making available whole foods, minimally processed foods, and selected processed foods. The resulting food preservation and processing infrastructure needs to be expanded.

A balanced and varied diet based on whole foods and natural ingredients can get us a long way and will hold all the essential components the human body needs. We could complement the food ecosystem with new processed food products with health benefits, for example, to increase diet adherence or adaptation to certain food products or food groups. Governments, the food industry, and academia have a large responsibility to promote adherence to healthy and sustainable diets and enable a FAM approach. Governments can contribute by providing health-promoting guidelines and legislation, industry can contribute by developing nutritious products and using responsible marketing to stimulate healthy choices, and academia can identify nutrition-health relationships and enlarge the scientific base of personalized dietary approaches.

In conclusion, we should better deploy the potential of food for promoting health and combating disease and acute and chronic health conditions. We consume foods multiple times per day, in contrast to most medications. Continuous, long-term exposure to its nutritional compounds has a huge potential to help prevent diet-induced diseases. Food should, therefore, become a key part of lifestyle medicine.

## Author contributions

The authors’ responsibilities were as follows – TD: conceptualized the study and acquired funding, did the project administration, performed the investigation and developed the methodology, developed the paper concept with key input of all coauthors, wrote the original draft of the paper with key input of all coauthors; TD, FB: did the visualization; TK, SA, MCEB, RMR, MG: performed critical review and editing; and all authors: read and approved the manuscript.

## Funding

This work was partly supported by the Swiss National Science Foundation (200020_200629). The funder was not involved in the study design, collection, analysis, interpretation of data, the writing of this article, or the decision to submit it for publication.

## Declaration of generative AI and AI-assisted technologies in the writing process

During the preparation of this work, the authors used Grammarly in order to improve spelling, grammar, and clarity of the text. After using this tool/service, the authors reviewed and edited the content as needed and take full responsibility for the content of the publication.

## Conflict of interest

The authors report no conflicts of interest. Note however that TK is affiliated with the Centre for Digital Health Interventions (CDHI), a joint initiative of the Institute for Implementation Science in Health Care, University of Zurich, the Department of Management, Technology, and Economics at ETH Zurich, and the Institute of Technology Management and School of Medicine at the University of St. Gallen. CDHI is funded in part by CSS, a Swiss health insurer, the Swiss growth-stage investor MTIP, and the Austrian health provider Mavie Next. TK was co-founder of Pathmate Technologies, a university spin-off company. However, Pathmate Technologies, CSS, MTIP, and Mavie Next were not involved in this study.
